# Biomechanical comparison of graft structures in anterior cruciate ligament reconstruction

**DOI:** 10.1007/s00167-016-4316-6

**Published:** 2016-09-16

**Authors:** Breck R. Lord, Hadi El-Daou, Bhushan M. Sabnis, Chinmay M. Gupte, Adrian M. Wilson, Andrew A. Amis

**Affiliations:** 1 The Biomechanics Group, Department of Mechanical Engineering, Imperial College London, London, SW7 2AZ UK; 2 Basingstoke and North Hampshire Hospital, Basingstoke, UK; 3 Musculoskeletal Surgery Group, Department of Surgery and Cancer, Imperial College London School of Medicine, Charing Cross Hospital, London, UK

**Keywords:** Anterior cruciate ligament, Anatomic ACL reconstruction, TriLink, Single bundle, Double bundle, Knee kinematics, Pivot shift, Robotics

## Abstract

**Purpose:**

Double-bundle (DB) anterior cruciate ligament (ACL) reconstruction may offer kinematic restoration superior to anatomic single bundle (SB), but it remains technically challenging. The femoral attachment site has the most effect on ACL graft isometry, so a simplified three-socket (3S) construct which still uses two sockets to cover the femoral ACL attachment is attractive. It was hypothesised that ACL reconstruction using three- and four-socket techniques would more closely restore native knee kinematics compared to anatomic two-socket (SB) surgery.

**Methods:**

Nine cadaveric knees were used to evaluate the kinematics of ACL-intact, ACL-deficient, anatomic SB, three-socket, and DB arthroscopic ACL reconstructions. Suspensory fixation was used, and grafts were tensioned to match the anterior draw of the intact knee at 20°. A six-degree-of-freedom robotic system measured knee laxity under 90 N anterior tibial force and rotational laxity under 5 N-m torque. Combined moments were applied to simulate the pivot-shift subluxation: 4 N-m internal rotation and 8 N-m valgus.

**Results:**

Significant differences between reconstructions were not found during anterior tibial loading, apart from SB being more lax than DB at 60° flexion. All reconstructions produced comparable laxity to the intact state, apart from SB at 60°. Significant differences between reconstructions were not found at any flexion angle during tibial internal/external applied torques. Under combined loading, DB produced significantly less laxity than SB constructs apart from anterior tibial translation at 0° and internal rotation at 45°. 3S and DB were comparable to the native knee throughout.

**Conclusion:**

Although 3S restored laxities to a similar extent to DB, significant superiority over SB surgery was not observed. Although statistically significant differences were found between SB and DB surgery during anterior tibial and simulated pivot-shift loading, both remained similar to the native knee. The clinical relevance is that this study did not support an ACL graft construct more complex than an anatomic single bundle.

## Introduction

The anatomy of the ACL is complex, with a multitude of small fascicular bundles twisting around each other during knee flexion [15], commonly simplified into two functional bundles: anteromedial (AM) and posterolateral (PL) based on their tibial attachment sites [13]. These contribute to knee stability in a specific pattern, depending on the knee flexion angle [1, 11, 39]. This pattern reflects the structure of the native ACL, functioning as groups of fibres which lengthen and slacken across the range of motion [16, 39].

The optimal method of ACL reconstruction is yet to be determined. Biomechanical [4, 25, 33, 34] and clinical [19, 21, 26, 42, 52] papers have demonstrated superior restoration of native knee kinematics or improved function after double-bundle (DB) surgery than single-bundle (SB) surgery; however, equivocal results have also been reported [14, 23, 24, 30, 32, 47, 48]. Double-bundle surgery is more complex, imposing greater technical demand, longer operative time, the possibility of tunnel convergence and notch impingement, and greater expense [3, 7, 8]. A DB reconstruction is defined in this paper as having two grafts, each with separate bone sockets at each end, forming a four-socket construct.

The femoral attachment site has the most effect on ACL graft isometry and tension with knee flexion [16, 27, 41], so a simplified graft construct which covers most of the femoral ACL attachment is attractive. Therefore, the purpose of this study was to examine the relative merits of a three-socket (3S) construct, with a V-configuration graft located into a single tibial tunnel and double femoral ACL graft tunnels [49]. Unlike previous work, the present study evaluated ACL reconstructions using cortical suspensory fixation, enabling different tensioning angles for each graft bundle. It was hypothesised that ACL reconstruction using three- and four-socket techniques would more closely restore native knee kinematics compared to anatomic single-bundle two-socket (SB) surgery, whilst 3S surgery would restore native knee kinematics to a similar extent as four-socket DB surgery. Although there have been many previous studies of ACL graft constructs, there has not been a study which has compared the relative biomechanical ability of each of the three constructs to restore native knee laxity.

## Materials and methods

### Specimen preparation

Nine fresh-frozen knees from donors with a mean age of 66 years (SD, ±7.6 years; range, 54–78 years; five female and four male; three left and six right), were used. They were screened for prior ACL injury, surgery, and soft tissue or bony disease. The semitendinosus and gracilis tendons were harvested in a retrograde manner via a 30-mm oblique incision over the pes anserinus. The femur was cut 190 mm from the joint line and the soft tissues resected from the proximal 80 mm. The tibia was cut 140 mm from the joint line and the soft tissue resected from the distal 60 mm. The fibula was transfixed to the tibia with a tri-cortical screw.

### Robotic system

**Fig. 1:**
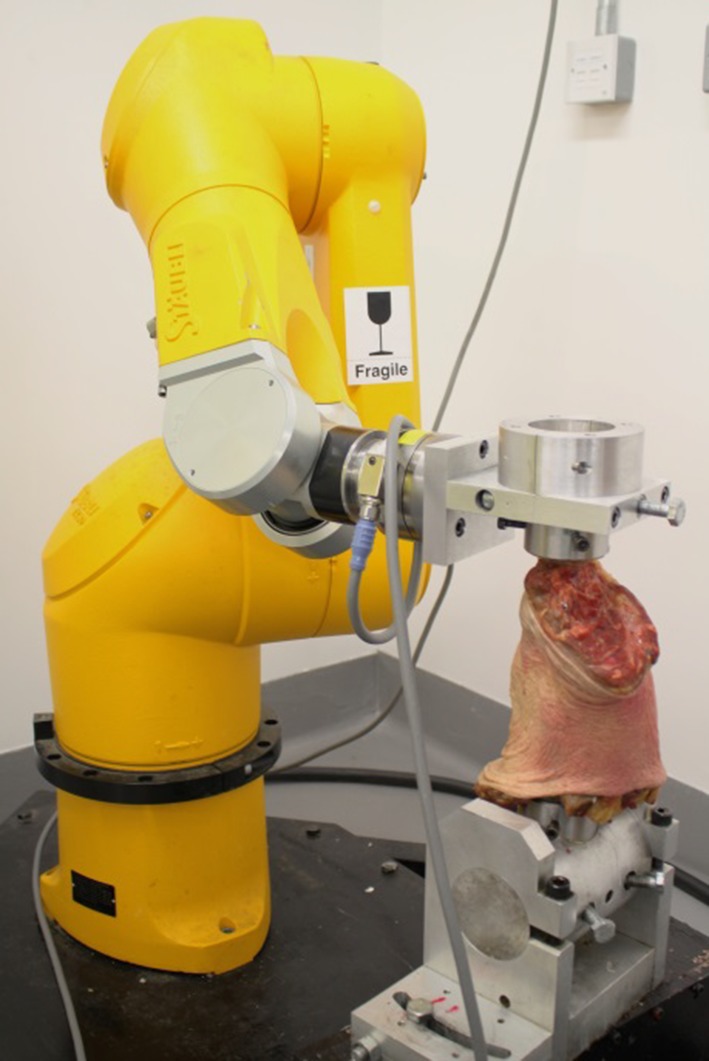
Robotic joint manipulator. The tibia was mounted within a custom fixture attached to a universal force–torque sensor (Omega 85, ATI Industrial Automation) affixed to the end effector of a robot (TX90, Stäubli Ltd, Switzerland), while the femur was mounted to the fixed base

Each knee was secured within a robotic biomechanical testing system, comprising of a six-degree-of-freedom (DOF) robotic manipulator (TX90, Stäubli Ltd, Switzerland), a six-axis universal force sensor (Omega 85, ATI Industrial Automation), with custom-designed tibial and femoral fixtures (Fig. 1). The force sensor had a resolution of 0.3, 0.3 and 0.4 N for *X*, *Y* and *Z* axis forces, respectively, and 0.01 Nm for *X*, *Y* and *Z* axis torques. The robotic system had a test–retest SD of ±0.10 mm and ±0.12° in translation and rotation between the bone mountings. The tibia was cemented into a 60-mm-diameter stainless steel pot using polymethylmethacrylate (Simplex Rapid, Kemdent, UK). The long axis of the cylinder was perpendicular to the joint surface in the coronal plane and parallel to the long axis of the bone in the sagittal plane. Zero degrees flexion was defined when 3.2-mm guide wires drilled postero-anteriorly through the tibia and femur at 70 and 100 mm away from the joint line, respectively, were parallel.

### Biomechanical testing

Maintaining 0° knee flexion, the system minimised the forces and torques in the remaining five DOFs and recorded a known starting point for the intact knee. From this point, the force sensor guided the passive path of knee flexion from 0° to 90° while minimising the five remaining forces and torques. Three cycles of flexion–extension were performed in order to minimise error from the inherent stress relaxation properties of soft tissue [18]. To quantify knee laxity, the robot held a chosen flexion angle on the passive path and a force/torque was imposed while the remaining 4 DOF were neutralised: 90 N for antero–posterior (AP) tibial translation, 5 Nm for internal/external rotation (IR/ER) and coupled moments of 4 Nm IR with 8 Nm of valgus to simulate the pivot-shift laxity [22]. The AP, IR, and ER laxities were evaluated at 0°, 30°, 60° and 90° of flexion [2, 11, 24, 47]. The simulated pivot shift (SPS) was performed at 0°, 15°, 30° and 45° of flexion [2, 9, 11, 22, 24, 47] and the tibial displacement divided into IR and anterior components.

### Surgical technique

ACL reconstructions were performed by a consultant soft tissue knee surgeon. Each reconstruction was performed first, second, and third on three occasions, eliminating bias. The ACL was resected arthroscopically, leaving 1-mm remnants to guide tunnel placement. A medial parapatellar arthrotomy was performed above the meniscus [14] to accurately visualise the ACL attachments and define the positions of the fibre bundles of the ACL. These were recorded relative to validated arthroscopic landmarks and used to guide subsequent socket placement [6, 20, 36]. The bone tunnels were repaired between reconstructions using a polyester paste by outside-in (femur) or inside-out (tibia) injection [23, 24].

Each reconstruction was performed using a translateral all-inside technique, using outside-in drilling (FlipCutter; Arthrex Inc, Naples, FL) and adjustable length cortical suspensory fixation (ACL TightRope RT, Arthrex) [28, 31, 43]. Grafts were constructed from either a single semitendinosus (SB and 3S) or semitendinosus plus gracilis (DB) tendon, pre-tensioned with 50 N for 10 min prior to deployment [2].

Adjustable suspensory fixation devices are closed-loop systems, and thus the resultant graft tension cannot be reliably determined from that applied externally during deployment, so a laxity-matching method was used. A Lachman test was performed on the intact knee: 90 N of anterior tibial force was applied and laxity quantified with a Rolimeter^®^ [37]. Starting at 30 N [24], the fixations were tensioned by 5 N increments until the laxity at 20° [29] was equivocal to the intact knee. The knee was subsequently cycled ten times from 0° to 120° and the tensioning procedure repeated. The arthrotomy was not closed, avoiding variability in soft tissue tension.

#### Anatomic double-bundle reconstruction

The semitendinosus and gracilis tendons were quadrupled to form individual grafts to replicate the AM and PL bundles, respectively. Each tendon was quadrupled through two ACL fixation devices (TightRope, Arthrex), then transfixed and secured with sutures (0-FiberWire, Arthrex) [31, 46]. The mean diameters of the AM and PL grafts were 8 and 7 mm, respectively. Guide wires were drilled through the centres of the native bundle attachments. Sockets were created to a depth of 25 and 30 mm on the femur and tibia, respectively. A 2-mm bony bridge was confirmed prior to graft deployment. Outside-in sutures were passed through the tunnels with passage of the PL followed by the AM graft. The cortical buttons were deployed onto the cortex under direct vision to avoid soft tissue impingement. The PL and AM bundles were tensioned at 0° and 60°, respectively [10].

#### Anatomic single-bundle reconstruction

A single semitendinosus tendon was quadrupled through two fixation devices and secured as previously described [46], producing a mean graft diameter of 8 mm. The graft tunnels were drilled through the centres of the ACL femoral and tibial attachments and their positions confirmed under direct vision. Graft tensioning was at 30° knee flexion [45].

#### Anatomic three-socket reconstruction

A single semitendinosus tendon was doubled and folded in half, producing a bifurcating graft as previously described [49]. The mean diameter of the single tibial bundle was 8 mm, the femoral AM and PL grafts were each 6 mm. Anatomic socket positions on the femur were identical to the DB technique whilst the anatomic SB mid-bundle position was used on the tibia [20]. The PL and AM bundles were tensioned at 0° and 60°, respectively [10].

### Tunnel position analysis

After testing, soft tissues were resected and the femur cut in the mid-sagittal plane at the intercondylar notch. True lateral photographs were taken of the medial aspect of the lateral femoral condyle and axial photographs of the tibial plateau (Single-lens reflex, Canon 100D). Overlay grids, as defined by Bernard et al. [5] and Tsuda et al. [44] were placed over the image of each femoral and tibial attachment, respectively, and the centres of the sockets were recorded (ImageJ 1.48, National Institute of Health, Bethesda, Maryland, USA).

### Study approval

The study protocol for obtaining, use and disposal of human tissue specimens received REC Wales approval: 12/WA/0196, permit number ICHTB HTA licence: 12,275.

### Statistical analysis

Based on data from a previous study [24], it was determined that for detection of a 2-mm change in anterior translation laxity with 80 % power at the 5 % level of significance, eight knee specimens would be required, based on the SD of the laxity being ±1.4 mm; nine were used, in case of any technical problem during the work. The kinematic data were analysed using a two-factor repeated-measures analysis of variance (RM-ANOVA). The two factors assessed were the state of the ACL and the flexion angle of the knee. Five dependent variables (tibial displacements) were evaluated with Bonferroni corrections: anterior translation, internal rotation (IR), external rotation, and anterior translation and IR under coupled loading during the SPS. Pairwise comparisons using a paired *t* test were performed where appropriate. The level of significance was set at *P* < 0.05 for a single comparison. Statistical analysis was performed in SPSS v 21, IBM Corp.

## Results

### Anterior tibial translation

**Fig. 2:**
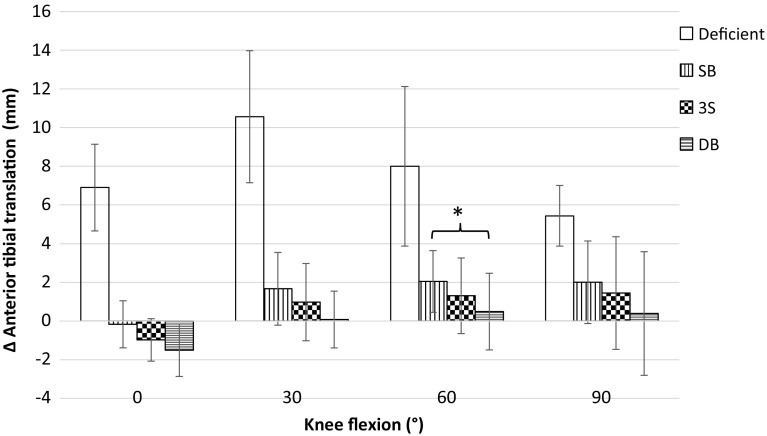
Change of anterior tibial translation from native knee laxity, in response to a 90 N anterior force (Mean ± SD). SB, single-bundle reconstruction; 3S, three-socket reconstruction; DB, double-bundle reconstruction. Significantly less laxity was found between all reconstructions and the deficient state except 3S in 90° of knee flexion

Significant increases in anterior tibial translation laxity were found from the intact to ACL-deficient states at all flexion angles. Similarly, all reconstructions were significantly less lax than the ACL-deficient knee at all flexion angles except 3S at 90°. Significant differences were not found between the intact state and any reconstruction, apart from SB being more lax at 60° of knee flexion. Similarly, significant differences were not found between reconstructions, apart from DB being significantly less lax than SB at 60° (Table 1; Fig. 2).

**Table 1:** Observed translational and rotational differences between knee states

Flexion angle	Translation at intact state (mm)	Differences from intact (mm)			
		ACL deficient	SB	TriLink	DB
Anterior tibial translation					
0°	4.0 ± 1.4	6.9 ± 2.2^i^	−0.2 ± 1.2^d^	−1.0 ± 1.0^d^	−1.5 ± 1.4^d^
30°	5.5 ± 2.0	10.6 ± 3.4^i^	1.7 ± 1.9^d^	1.0 ± 2.0^d^	0.1 ± 1.5^d^
60°	5.3 ± 1.7	8.0 ± 4.1^i^	2.0 ± 1.6^d,i,r^	1.3 ± 2.0^d^	0.5 ± 2.0^d,r^
90°	5.1 ± 2.2	5.4 ± 1.6^i^	2.0 ± 2.1^d^	1.5 ± 2.9	0.4 ± 3.2^d^

Flexion angle	Translation at intact state (mm)	Difference from intact (mm)			
		ACL deficient	SB	TriLink	DB
Simulated pivot shift: anterior tibial translation					
0°	2.2 ± 1.6	3.6 ± 2.0^i^	0.2 ± 1.3^d^	−0.8 ± 1.5^d^	−1.5 ± 1.2^d,i^
15°	2.8 ± 2.2	4.6 ± 2.0^i^	0.8 ± 1.6^d,r^	−0.0 ± 1.6^d^	−0.8 ± 0.9^d,r^
30°	3.4 ± 2.8	4.5 ± 2.3^i^	1.0 ± 1.7^d,r^	0.2 ± 1.8^d^	−0.6 ± 1.2^d,r^
45°	2.8 ± 3.0	3.8 ± 3.3^i^	1.1 ± 1.7^r^	0.6 ± 1.8	0.1 ± 1.59^d,r^

Flexion angle	Rotation at intact state (°)	Difference from intact (°)			
		ACL deficient	SB	TriLink	DB
Simulated pivot shift: internal tibial rotation					
0°	10.4 ± 2.8	3.5 ± 2.1^i^	0.8 ± 1.1^d,r^	−0.2 ± 1.5^d^	−1.0 ± 1.2^d,r^
15°	16.3 ± 6.1	2.8 ± 2.1^i^	1.6 ± 1.5^r^	0.6 ± 1.5	−0.2 ± 1.0^d, r^
30°	19.0 ± 8.5	1.6 ± 1.6^i^	1.3 ± 1.2^r^	0.7 ± 1.0	0.1 ± 0.6^r^
45°	19.8 ± 10.0	1.0 ± 1.0^i^	1.0 ± 1.0	0.5 ± 0.9	0.5 ± 0.7

Flexion angle	Rotation at intact state (°)	Difference from intact (°)			
		ACL deficient	SB	TriLink	DB
Internal tibial rotation					
0°	10.6 ± 3.1	3.3 ± 1.7^i^	0.5 ± 1.4^d^	−0.9 ± 1.4^d^	−1.4 ± 1.6^d^
30°	19.2 ± 9.2	1.8 ± 1.4^i^	0.7 ± 0.8	0.4 ± 0.9	−0.1 ± 0.7^d^
60°	18.6 ± 9.7	1.1 ± 0.6^i^	0.6 ± 0.6	0.6 ± 0.7	0.5 ± 0.8
90°	18.4 ± 10.0	0.8 ± 0.3^i^	0.6 ± 0.6	0.7 ± 0.8	0.6 ± 0.8

Flexion angle	Rotation at intact state (°)	Difference from intact (°)			
		ACL deficient	SB	TriLink	DB
External tibial rotation (°)					
0°	12.1 ± 4.7	0.5 ± 0.5^i^	0.0 ± 0.9	−0.3 ± 0.7	−0.1 ± 0.8
30°	20.4 ± 9.2	0.6 ± 0.4^i^	0.1 ± 0.8	−0.1 ± 0.6	0.3 ± 1.1
60°	21.6 ± 10.9	0.8 ± 0.6^i^	0.2 ± 0.9	−0.0 ± 0.5^d^	0.1 ± 0.7
90°	21.2 ± 9.5	0.6 ± 0.4^i^	0.1 ± 0.9	−0.0 ± 0.5^d^	−0.1 ± 0.8^d^

Values are expressed as mean ± standard deviation. Measurements are reported relative to the intact state of each knee. SB, single-bundle reconstruction; 3S, three-socket reconstruction; DB, double-bundle reconstruction. Statistically significant difference from ^i^ intact state, ^d^deficient state and ^r^between reconstructions (*P* < 0.05)

### Internal/external tibial rotation

Significant increases in IR and ER were observed from the intact to the ACL-deficient states at all flexion angles tested. Conversely, significant differences in IR and ER were not found between the intact state and any reconstruction, or between reconstructions, at any flexion angle. All reconstructions allowed significantly less IR than the deficient state at 0°, only DB surgery did so at 30°, whilst no reconstruction had significantly less rotation than the deficient knee at 60° and 90° (Table 1).

### Simulated pivot shift

**Fig. 3:**
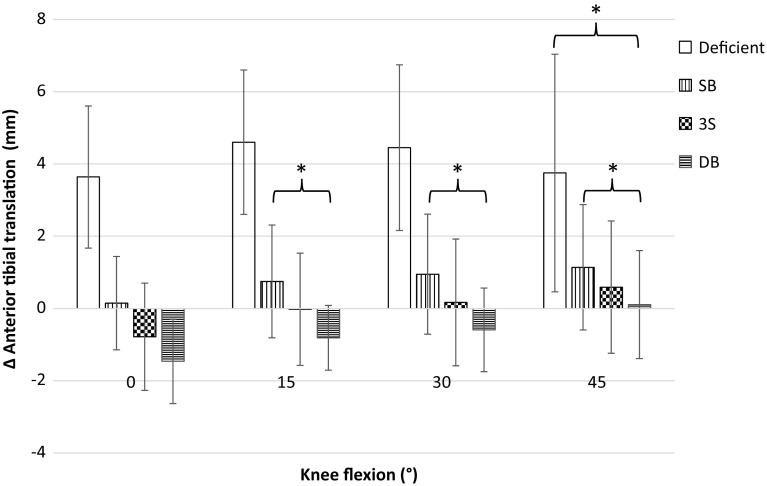
Change of coupled anterior tibial translation from native knee laxity in response to combined 4 N-m of internal tibial and 8 N-m of valgus torques (Mean ± SD). SB, single-bundle reconstruction; 3S, three-socket reconstruction; DB, double-bundle reconstruction. Significantly less laxity was found between all reconstructions and the deficient state except for SB and 3S in 45° of knee flexion

Significant increases in coupled anterior translation were found from the intact to the ACL-deficient state at all flexion angles tested. Similarly, all reconstructions were significantly less lax than the ACL-deficient knee at all flexion angles apart from SB and 3S at 45°. Significant differences in coupled anterior translation were not found between the intact state and any reconstruction at any flexion angle. No significant difference was observed between SB and 3S, or 3S and DB, at any flexion angle. DB was significantly less lax than SB at 15°, 30° and 45°; however, both were similar to the intact knee (Table 1; Fig. 3).

**Fig. 4:**
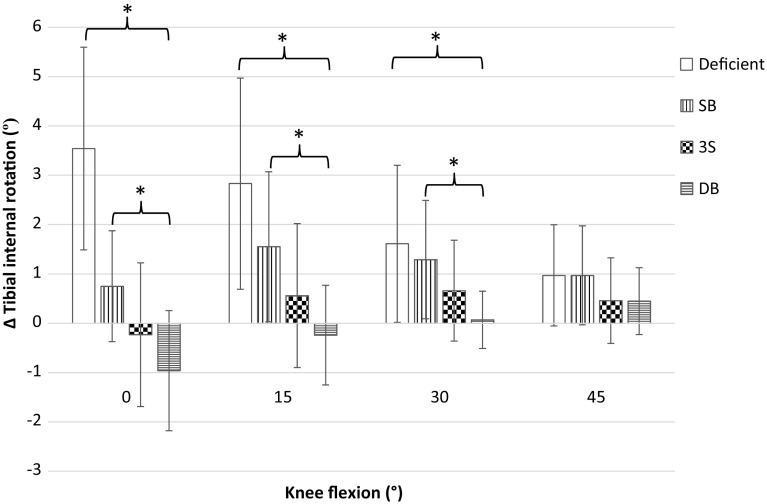
Change of internal tibial rotation from the laxity of the native knee in response to combined 4 N-m of internal tibial and 8 N-m of valgus torques (Mean ± SD). SB, single-bundle reconstruction; 3S, three-socket reconstruction; DB, double-bundle reconstruction

Significant increases in IR under simulated pivot-shift loading were found from the intact to the ACL-deficient state at all flexion angles. All reconstructions were significantly less lax than the ACL-deficient knee at 0°, DB was significantly less lax at 15° and 30° and all reconstructions were comparable with the deficient knee at 45°. Significant differences in IR were not found between the intact state and any reconstruction during coupled loading at any flexion angle. No significant difference was observed between SB and 3S at any flexion angle. Whilst DB was significantly less lax than SB at 0°, 15° and 30°, DB and 3S laxities were comparable (Fig. 4).

**Fig. 5:**
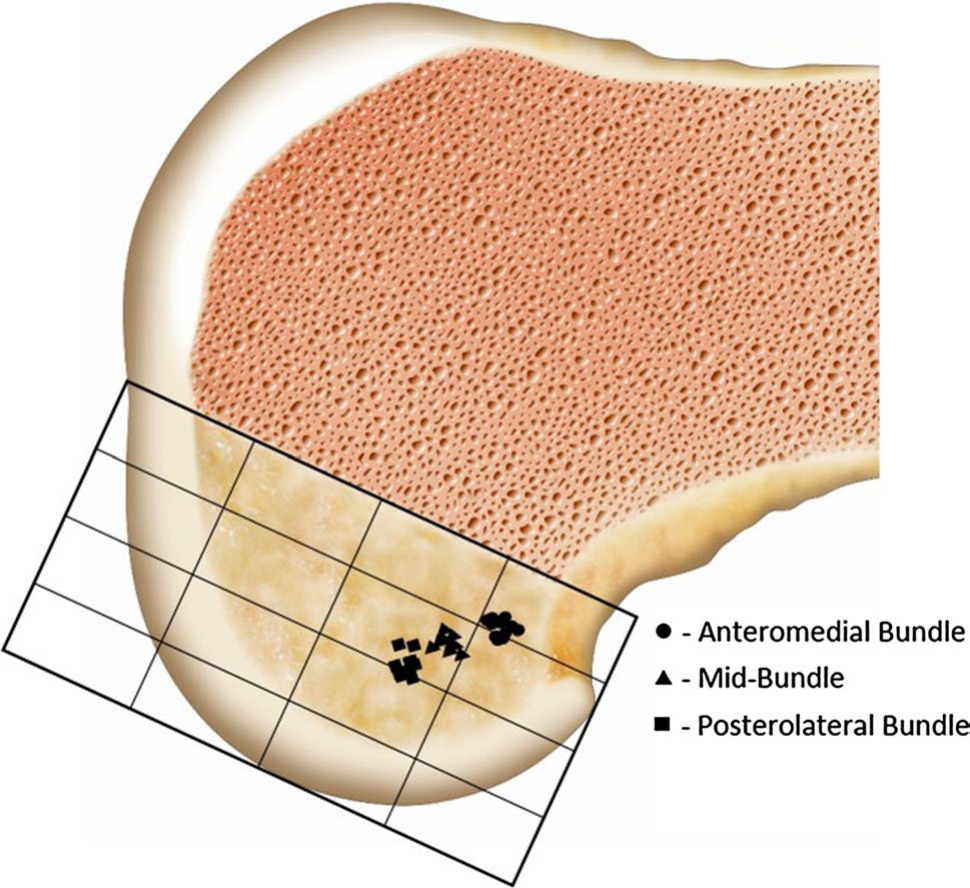
Post hoc analysis of the three socket positions on the femur

**Fig. 6:**
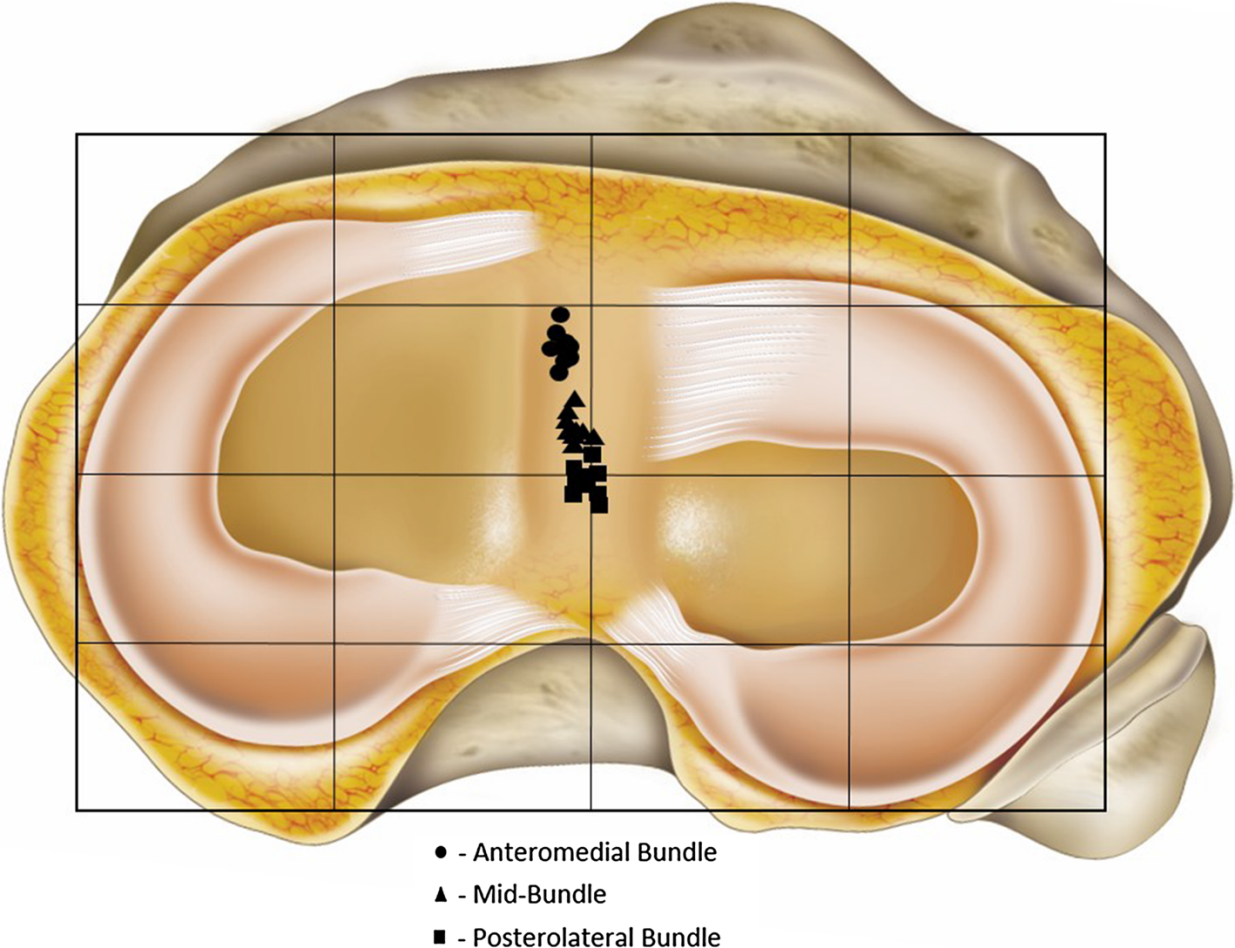
Post hoc analysis of the three socket positions on the tibia

Socket positions after SB, 3S, and DB surgery are listed in Table 2 and shown in Figs. 5 and 6.

**Table 2:** Mean socket positions following post hoc analysis

		AM (%)	MB (%)	PL (%)
Femur	Proximal–distal	21 ± 2	29 ± 2	34 ± 2
	Anterior–posterior	23 ± 3	35 ± 2	47 ± 4
Tibia	Medial–lateral	47 ± 1	48 ± 1	49 ± 1
	Anterior–posterior	32 ± 3	44 ± 2	51 ± 3

On the femur, measurements start from 0 % at the proximal, anterior edge of a superimposed grid aligned with the roof of the femoral notch—the zero position was deep and high in the notch (Fig. 5). On the tibia, measurements started at 0 % from the anteromedial corner of the grid fitted to the tibial plateau (Fig. 6)

*AM* anteromedial bundle, *MB* mid-bundle, *PL* posterolateral bundle

## Discussion

The most important finding of this study is that, contrary to the hypothesis, a bifurcating graft with three bone sockets (3S) did not restore native knee laxities significantly more closely to normal than a single-bundle (SB) ACL reconstruction. Laxities after 3S and DB surgery were comparable, with the DB reconstruction providing significantly less laxity than SB surgery under the coupled moments of the simulated pivot shift. Tensioning the ACL grafts to match a quantified Lachman test of the intact knee also offered restoration of intact laxity of IR and the SPS for all reconstructions.

This is the only biomechanical study comparing the kinematics of anatomic single- and multi-bundle ACL reconstructions with total suspensory fixation. This adjustable fixation allowed a laxity-matching graft tensioning protocol to be used, akin to minimising the side-to-side difference clinically; it allowed the knees to be restored close to intact behaviour with all three graft configurations. Previous biomechanical studies have applied a range of pre-determined graft tensions with varied results, often reporting significantly more laxity than the native knee [2, 17, 51], especially during IR and the SPS. This emphasises the value of quantifying the laxity of the ‘intact’ contralateral knee per-operatively, and subsequent graft tensioning, towards optimising the control of knee rotation at time zero.

Some studies have previously evaluated three-socket grafts of different graft type, configuration or fixation to that of the present study [23, 35, 47, 48]. Yagi et al. [47] reported that three-tunnel surgery more closely restored intact anterior laxity than an SB reconstruction at 0° and 30° and in situ graft forces from 0° to 60°; both reconstructions failed to restore native anterior restraint from 0° to 60° of flexion. Conversely, Yamamoto et al. [48] found no significant difference in anterior laxity between three-tunnel and laterally placed SB except at higher angles of flexion where SB was superior. Petersen et al. [35] compared a similar three-tunnel construct to DB surgery, reporting significantly less anterior laxity at 0° and 30° for the latter. In contrast, the present study found no significant difference between 3S and DB surgery at any angle of flexion; only the SB at 60° had significantly greater laxity. Kim et al. [23] compared the kinematics of four anatomic quadriceps ACL grafts: three-bundle SB with two femoral and one tibial sockets, three-bundle SB with two tibial and one femoral sockets, and DB reconstruction. All multi-bundle reconstructions were similar to the intact with significantly less anterior laxity than SB at 60° and 90°, whilst no difference was seen between the SB and deficient states at these flexion angles. In the present study, the SB was tensioned at 30° rather than in extension; this may have contributed to an improved performance at greater angles of knee flexion.

A number of studies have reported coupled tibial displacements in response to SPS loading. Kim et al. [23] found no differences in ATT between any knee state under simulated pivot shift, but found significantly lower forces in the SB construct than the intact ACL from 0° to 30° and significantly higher forces in DB and one-femur/two-tibia tunnel constructs at 15° and 30°, respectively. In contrast, this study found significant increases of coupled motion between the intact and ACL-deficient states at extension and early flexion, similar to previous work [14, 48]. Yagi et al. [47] found significantly less coupled ATT during the SPS after two-femur/two-tibia reconstruction than SB surgery, but it remained significantly more than the intact state. Petersen et al. [35] reported a similar superiority of DB over a two-femur/one-tibia graft, with significantly less ATT at 0° and 30° whilst also remaining significantly more lax than the intact state. Similarly to Yamamoto et al. [48], this study found that anatomic SB and a two-femur/one-tibia graft (3S) were able to restore ATT to values similar to the intact state. DB surgery had a tendency to over-constrain the joint in extension, which has been reported previously [25].

The present study further examined the IR component of the simulated pivot shift, and all reconstructions were comparable to the intact state. In contrast to Kondo et al. [25], significant increases in IR were seen between the intact and ACL-deficient states at all flexion angles. This remained true for SB and 3S, whilst DB surgery maintained significantly less laxity than the deficient state at 0° and 15°, as well as the SB state from 0° to 30°, near the angle at which the pivot shift occurs [12]. These findings were consistent with previous work suggesting that SB and two-femur/one-tibia 3S grafts produced sufficient control at 15°, but became less efficacious with increased knee flexion [48].

There are several limitations of the present study. Firstly, the specimens were 66 ± 8 years old, higher than the patient group who experience ACL rupture, but comparable to similar cadaveric studies [24, 43]. The clinical pivot shift is a dynamic examination through a range of motion. We were unable to replicate this using a single robotic manipulator, and the combined moments were imposed at a static flexion angle, therefore this and other studies [14, 23, 47] have not mimicked the in vivo kinematics but only the coupled laxities. These results represent time-zero data and do not account for ACL graft and other soft tissue changes during rehabilitation. This may be particularly relevant in the context of the initially over-constrained knee that may settle to a clinically effective reconstruction. Finally, there was no muscle loading, so these results reflect passive restraints, as in clinical laxity testing. The advantages of the study design, however, included the ability to perform three ACL reconstructions in each knee, thus allowing the within-specimen repeated-measures statistical analysis to discern their relative performances without inter-specimen variability.

The clinical relevance of this work relates to the choice of ACL reconstruction, and how that choice is affected by differences in the resulting laxity of the knee, among the SB, 3S, and DB constructs in this study. This study suggests equivalence for SB, 3S, and DB ACL reconstructions in their control of tibial anterior translation and rotation. This supports the use of a correctly positioned SB graft; while there was a consistent trend for reduced laxity with 3S over SB, it was not significant during any single comparison. Yasuda et al. [50] reported significantly better control of ATT and the pivot shift 2 years after DB compared to SB surgery. In keeping with the present study, the two-femur/one-tibia graft produced comparable laxity to the DB but not significantly less than the SB, despite a reported increase in stability with complete attachment site restoration [40]. The three-socket 3S surgery is less complicated than DB surgery yet produced comparable results; it preserves bone stock and spares gracilis, which is beneficial in the context of multi-ligament injury. This study suggests that it could be considered as an alternative to DB surgery. The literature reports a wide variety of graft tensions during ACL reconstruction [2, 17, 51], and although tensioning devices are available, no definitive clinical benefit has been reported [38]. This study suggests that quantifying the Lachman test of the uninjured knee and using this as a tensioning guide closely restores native knee laxity.

## Conclusion

Although three-socket reconstruction restored laxities to a similar extent to DB, no superiority over SB surgery was observed. Statistically significant differences were found between SB and DB surgery during anterior tibial and SPS loading during early flexion; however, both remained similar to that observed in the native knee. Single-bundle surgery, with anatomic tunnel position and the laxity matched to the native knee by use of adjustable fixation, provided clinically equivalent control of rotation compared with the intact knee.
